# Branched Motifs Enable Long-Range Interactions in Signaling Networks through Retrograde Propagation

**DOI:** 10.1371/journal.pone.0064409

**Published:** 2013-05-31

**Authors:** Tharmaraj Jesan, Uddipan Sarma, Subhadra Halder, Bhaskar Saha, Sitabhra Sinha

**Affiliations:** 1 The Institute of Mathematical Sciences, Chennai, India; 2 Health Physics Division, Bhabha Atomic Research Centre, Kalpakkam, India; 3 National Centre for Cell Science, Ganeshkhind, Pune, India; International Center for Genetic Engineering and Biotechnology, India

## Abstract

Branched structures arise in the intra-cellular signaling network when a molecule is involved in multiple enzyme-substrate reaction cascades. Such branched motifs are involved in key biological processes, e.g., immune response activated by T-cell and B-cell receptors. In this paper, we demonstrate long-range communication through retrograde propagation between branches of signaling pathways whose molecules do not directly interact. Our numerical simulations and experiments on a system comprising branches with JNK and p38MAPK as terminal molecules respectively that share a common MAP3K enzyme MEKK3/4 show that perturbing an enzyme in one branch can result in a series of changes in the activity levels of molecules “upstream” to the enzyme that eventually reaches the branch-point and affects other branches. In the absence of any evidence for explicit feedback regulation between the functionally distinct JNK and p38MAPK pathways, the experimentally observed modulation of phosphorylation amplitudes in the two pathways when a terminal kinase is inhibited implies the existence of long-range coordination through retrograde information propagation previously demonstrated in single linear reaction pathways. An important aspect of retrograde propagation in branched pathways that is distinct from previous work on retroactivity focusing exclusively on single chains is that varying the type of perturbation, e.g., between pharmaceutical agent mediated inhibition of phosphorylation or suppression of protein expression, can result in opposing responses in the other branches. This can have potential significance in designing drugs targeting key molecules which regulate multiple pathways implicated in systems-level diseases such as cancer and diabetes.

## Introduction

**Figure 1:**
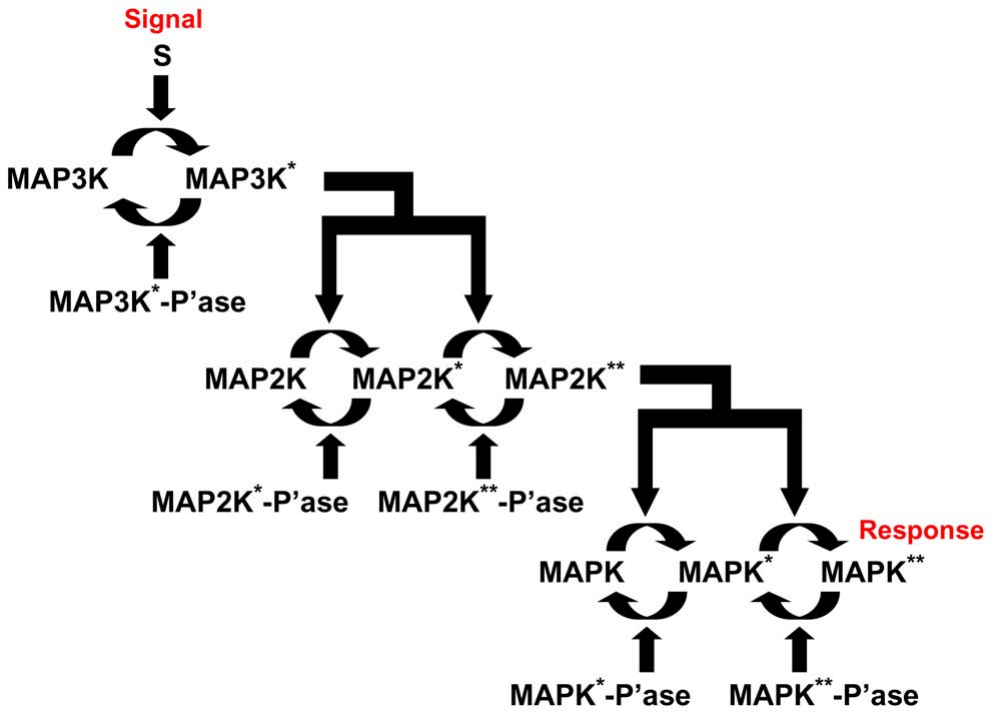
Schematic representation of the mitogen-activated protein kinase (MAPK) cascade. Activation through phosphorylation of the substrate kinase at each step is mediated by the upstream kinase, the single and doubly phosphorylated states of the substrate being denoted by the superscripts “*” and “**”, respectively. Activated kinases are eventually dephosphorylated into their inactive forms by the corresponding phosphatases (indicated by the suffix “-P’ase”). Activity in the three-component pathway is initiated by a signal S regulating the activation of the MAPK kinase kinase (MAP3K). Activated MAP3K controls the activation of MAPK kinase (MAP2K), which in its turn regulates the activation of MAPK. Note that unlike the single phosphorylation of MAP3K, both MAP2K and MAPK require double phosphorylation in order to become active, i.e., capable of acting as the enzyme for the corresponding downstream substrate protein. The eventual response of the cascade is quantified by the concentration of activated MAPK, which can be used in initiating transcription, activating other protein molecules, etc.

The intra-cellular signaling machinery is an extremely large and complex network that is best understood in terms of interactions between *modules*, i.e., well-defined sub-networks of interacting proteins. Such modules, often associated with specific functions, are distinguished by a relative level of insulation from the activity of other molecules [1]. However, as they are connected via the network, functions of individual modules can affect that of others in various complicated ways. The resulting adaptation of response to different circumstances allows the same module to be reused in many distinct contexts. Investigating the dynamical response of a basic module to various perturbations may give us a deeper understanding of its global role in the overall functioning of the network. Such a standard signaling module, found in all eukaryotic cells, is the three component mitogen-activated protein kinase (MAPK) cascade involved in many critical cellular functions including cell cycle control, stress response, differentiation, growth, etc. [2]–[4]. It is affected in many diseases including cancer, as well as, immunological and degenerative syndromes and is an important drug target [5]. The well-understood linear cascade involves the regulation by an input signal of the activity of a MAPK kinase kinase (MAP3K) that controls the activation of a MAPK kinase (MAP2K) which in turn controls the activity of a MAPK (Fig. 1). The end-result of MAPK activation is to initiate transcription or to stimulate the activity of other kinases [6].

However, such linear or chain-like reaction schemes imply a rigid relation between stimulus and response, precluding the possibility of the system switching to a different response for the same signal under altered circumstances. This adaptive ability is essential for survival of a cell in a noisy and dynamic environment. In fact, many linear cascades are actually part of *branched* pathways. For example, the MAP3K MEKK-1/2/3 are known to be capable of activating both JNK and ERK pathways [7]. The MAP3K MEKK-1 has also been seen to activate both the JNK and p38 pathways in the T-cell receptor signaling network [8], as well as, in the network downstream of the B-cell antigen receptor [9]. Such a design provides the cellular signaling apparatus the complexity necessary to allow integration of several signals and to regulate multiple functions at the same time [6], [10]. In particular, divergent signaling, where the activity of one molecule provides the input to multiple linear cascades allow the same external signal to generate different possible responses, the actual output being decided by the internal cellular context [6]. Such differential regulation can be achieved through reciprocal inhibition between the different branching pathways resulting in the eventual dominance of one of the possible responses. Thus, the ubiquity of branched pathway modules in the signaling network may be a consequence of the adaptability they provide to a cell in terms of making context-dependent choice between different responses.

In this paper we show that, in addition to having more flexibility compared to linear cascades, branched pathways allow complex long-range coordination of activity even in the absence of any direct long-range connections (i.e., reactions) between molecules. This allows complex dynamical control in conjunction with economy of wiring, measured by the total number of different possible chemical reactions, in the intra-cellular signaling network. Restricting the total number of links is desirable in a complex system, as high connectivity in a network can reduce the specificity of the response as many different signals can elicit the same activity [11], [12]. We show that reciprocal control between parallel reaction cascades stimulated by a common signal can occur in the absence of any coupling between the reactants in the different pathways. This non-local control takes place through retrograde information propagation previously demonstrated for linear reaction cascades [13], [14], in contrast to the classic forward flow from the input signal to the response of the terminal molecular species (e.g., from MAP3K to MAP2K to MAPK). Inhibition of the terminal molecule of one pathway in such a system can initiate a series of perturbations which travel upstream all the way to the molecule at the branch-point and from there, downstream along the other parallel pathways, altering the activity of several molecules all over the network. These predictions have been experimentally verified by us in macrophage cells, where blocking JNK phosphorylation is observed to bring about amplification of p38MAPK activity, and vice versa.

The possibility of reverse communication between components of the intracellular signaling network implies that branched pathways cannot be considered as simple open-loop circuits. Instead, retrograde propagation effectively implement closed-loop or feedback circuits, where perturbing one of the “output” elements can result in changes at the branch-point (at the “input”-end of the module), and thus, eventually to all other outputs of the module. Although the existence of implicit feedbacks in activation-deactivation reaction pathways have been shown earlier in the case of a simple linear MAPK cascade [13], [14], the consequences of combined forward and retrograde propagation of information in complex signaling network motifs have so far been unexplored. Our results reveal that even in absence of explicit long-range connections, local perturbations in one section of a signalling network can have systems-level consequences.

## Results

**Figure 2:**
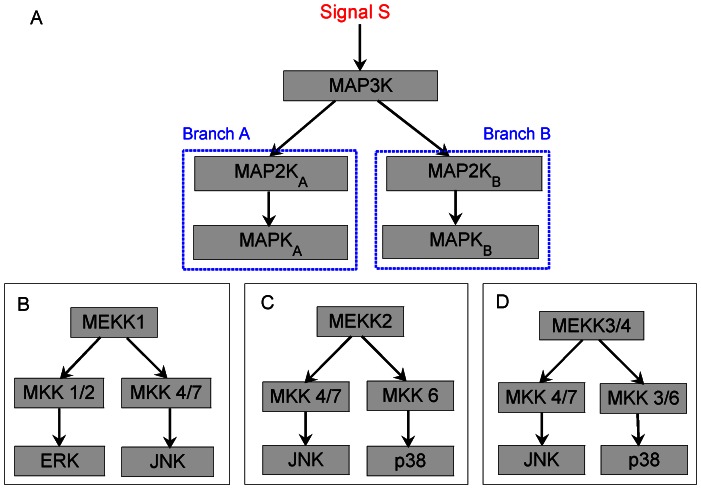
The branched MAPK cascade network motif. (A) A schematic diagram representing a simple branched cascade with two parallel pathways that is seen in many experimental systems, e.g., the T-cell receptor network [8]. The initial signal S activates a common MAP3K that can phosphorylate two different types of MAP2K molecules, viz., MAP2K*_A_* and MAP2K*_B_*. Each MAP2K type activates a particular type of MAP kinase, MAPK*_A_* and MAPK*_B_*, respectively. Specific examples of branched MAPK cascade motifs obtained from the experimental literature are shown in (B–D). They correspond to systems with the specific MAP3K (the branching point of the motif) being (B) MEKK1 that activates both MKK1/2 [15] and MKK4/7 [16], [17], (C) MEKK2 that activates both MKK4/7 [18], [19] and MKK6 [19], and (D) MEKK3/4 that activates both MKK4/7 [18]–[22] and MKK3/6 [19]–[21].

**Figure 3:**
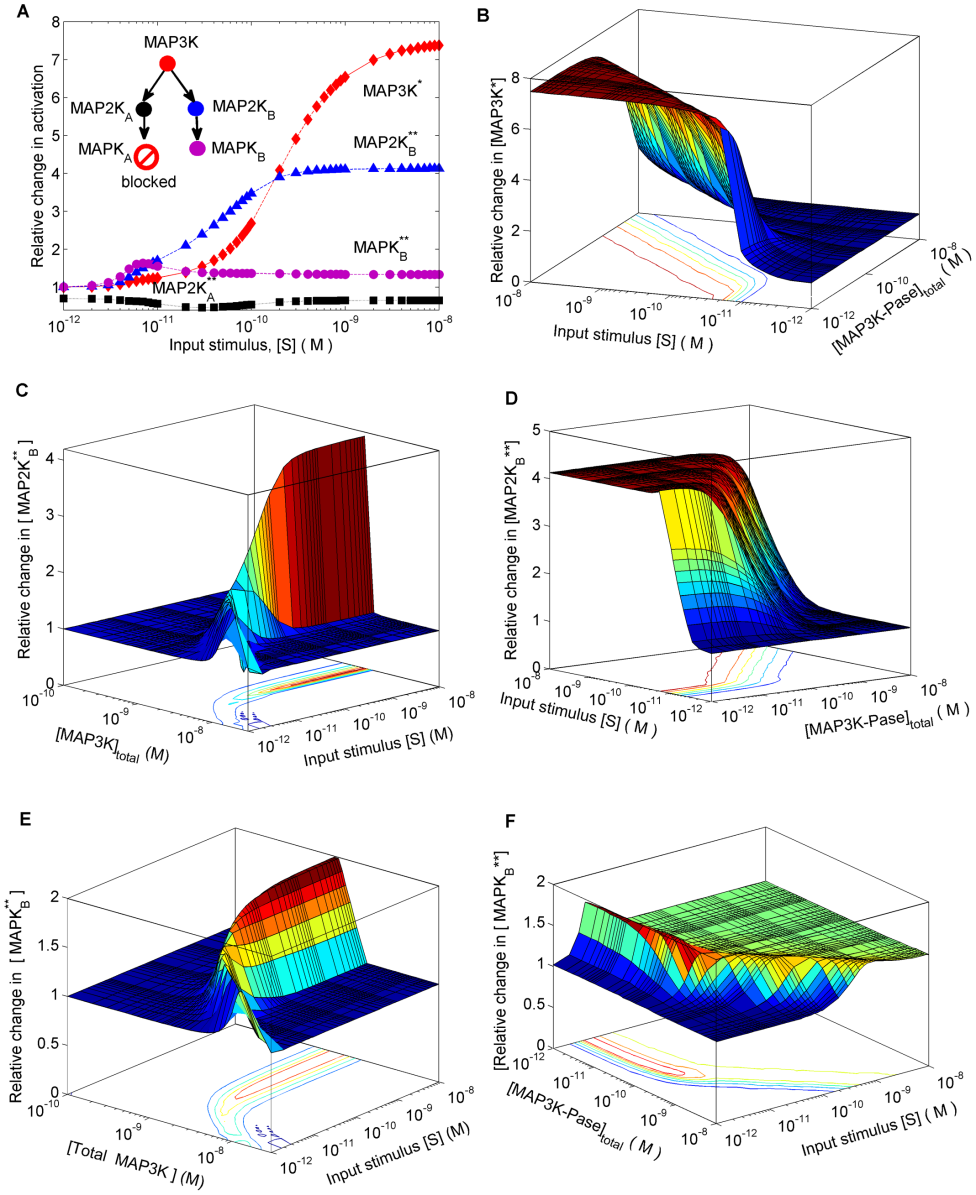
Amplification of response in unperturbed branch through retrograde propagation of information in a branched motif. (A) Relative change in the response of different molecular species in branches A and B as a function of signal strength, when phosphorylation of terminal kinase MAPK*_A_* in branch A is inhibited. Inset shows a schematic diagram of the branches, with the “stop” sign indicating blocking of activation of MAPK in branch A. (B–F) The perturbation results in amplification (relative to the unperturbed condition) of the concentrations of (B) MAP3K* (D) MAP2K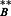 and (F) MAPK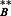, shown as a function of signal strength and concentration of the phosphatase MAP3K-Pase. Relative increase in concentrations of (C) MAP2K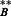 and (E) MAPK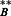 as a result of the perturbation are also shown as a function of signal strength and the total concentration of MAP3K.

We study in detail the model of a branched network motif shown in Fig. 2 and Fig. 3 (A): a MAP3K-MAP2K*_A,B_*-MAPK*_A,B_* cascade where the MAP3K, upon activation by an input signal (stimulus) S, phosphorylates two different types of MAP2K (designated MAP2K*_A_* and MAP2K*_B_* respectively). The doubly phosphorylated MAP2Ks in turn act as enzymes for the phosphorylation of the respective MAPK molecules designated as MAPK*_A_* and MAPK*_B_* respectively (see Materials and Methods and Text S1). When the product formation rate of MAPK*_A_*, 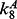 (see Text S1) is suppressed, we observe noticeable changes in the phosphorylation levels of the other kinases in the system even though the affected molecule is at the downstream terminus of the cascade. This is somewhat counter-intuitive as we normally expect information to only flow “down” the cascade from MAP3K to MAPK. In contrast, here were see that information about the suppression of MAPK activity can also travel in the opposite direction, i.e., “up” the cascade from MAPK to MAP3K, a phenomenon that we term as “retrograde” propagation. In experiments, preventing MAPK phosphorylation can be realized by blocking the ATP binding site of the target kinase. Fig. 3 (A) shows the relative change in the response of the system as a result of preventing activation of MAPK*_A_* (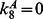), in terms of the relative increase or decrease in the steady-state concentrations of kinases in different branches compared to the unperturbed values. We observe that the perturbation reduces the activation of MAP2K*_A_* but all kinases in the unperturbed branch B, as well as, the MAP3K common to both branches, exhibit significant amplification in their phosphorylation. The modulation of kinase activity in the unperturbed branch in the absence of any direct connection between the molecules in the two branches reveals an implicit cellular mechanism for long-range communication in signaling networks through retrograde propagation. When 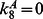, the concentration of free MAP2K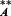 is reduced as most of it is trapped in the enzyme-substrate kinase (ESK) complex MAP2K
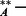
MAPK*_A_* from which it can be released only at the relatively slow rate of complex unbinding (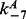). As more MAP2K*_A_* molecules are phosphorylated and end up bound in the above complex, this in turn reduces the concentration of free MAP2K*_A_*. The decrease in free MAP2K*_A_* results in MAP2K*_B_* gaining relatively more access to MAP3K that enhances the phosphorylation of branch B kinases.

As the phosphorylation of the MAP3K at the branch-point is modulated by the strength of the external signal S while the dephosphorylation of MAP3K* is mediated by the concentration of its phosphatase MAP3K-Pase, we examine how different combinations of signal dose and phosphatase concentrations affect the extent of retrograde propagation in the system. Fig. 3 (B) shows the coupled effect of the strength of the input stimuli S and the concentration of [MAP3K-Pase] on the relative change in activity of MAP3K when the activation of MAPK*_A_* is blocked (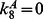). The enhanced concentration of MAP3K* results in a corresponding increase in activity of the B branch kinases, MAP2K*_B_* (Fig. 2 D) and MAPK*_B_* (Fig. 3 F). The amount of total MAP3K available also affects the relative increase in activity of the unperturbed branch as a result of the blocking of MAPK*_A_* phosphorylation. As seen in Figs. 3 (C) and 3 (E), there is an optimal range of total MAP3K concentration, [MAP3K], where the largest relative increase in the activity of MAP2K*_B_* and MAPK*_B_* is observed for a given signal strength. In particular, when [S] is varied over 10^−12^–10^−8^ M, i.e., the physiologically plausible range of values, the largest relative change in [MAP2K_B_**] and [MAPK_B_**] occurs between between [MAP3K] = 10^−9^–10^−8^ M.

The perturbation we have discussed above corresponds to complete blocking of MAPK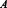 phosphorylation. We also examine the effect of a *graded* perturbation where the product formation rate of the ESK complex (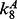) is decreased but remains finite (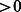). As the magnitude of this perturbation is increased (i.e., 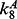 is decreased to even lower values), it will approach the situation described earlier: complete absence of MAPK*_A_* activity. We note that another class of perturbations may also superficially exhibit a similar nature, viz., gradually reducing the total concentration of MAPK*_A_* which will affect the reaction flux by reducing the formation of the ESK bound complex. For this case also, as the magnitude of perturbation is increased, the concentration of activated MAPK*_A_* steadily approaches zero. These two types of interventions correspond respectively to (i) using an ATP inhibitory agent targeting the ATP binding site, and (ii) using siRNA to block the expression for the gene coding for MAPK*_A_*, with both interventions having less than 100% efficiency.

**Figure 4:**
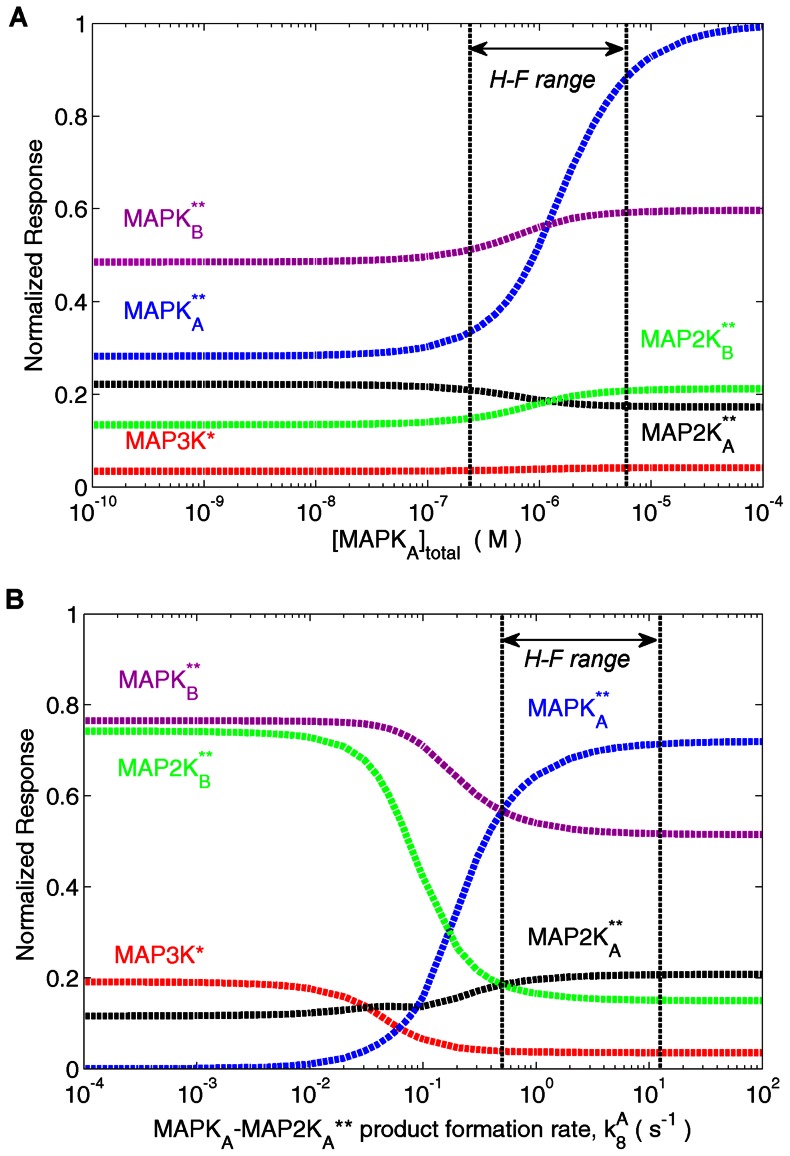
The branched MAPK cascade responds differently to to distinct perturbations which inhibit the activation of a terminal kinase in one branch. The normalized response, i.e., the ratio of activated to total kinase concentration, of different molecular species in the reaction cascade when (A) the total concentration of MAPK*_A_* is varied and (B) when the product formation rate (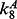) of MAPK*_A_* is varied shows opposing behavior in the activity of molecules in the unperturbed B branch, although both types of perturbations have the same functional goal of decreasing the activation of MAPK*_A_*. As [MAPK*_A_*]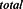 is decreased, both MAPK*_B_* and MAP2K*_B_* decrease in activity. However, when the product formation rate of MAPK*_A_* is decreased, the activity of both MAPK*_B_* and MAP2K*_B_* are increased. The activity of the kinase MAP3K which forms the branch-point also shows different response to the two perturbations unlike the result for single-branch cascades: in (A), the activity is unchanged, whereas in (B), the activity of MAP3K increases, on decreasing the activation of MAPK*_A_*. Note that the curves corresponding to MAPK*_A_* and MAPK*_B_* intersect in (A) when [MAPK*_A_*]*_total_* = [MAPK*_B_*]*_total_* = 1.2 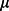
M and they intersect in (B) when the product formation rates for MAPK*_A_* and MAPK*_B_*, i.e., 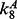 and 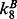 respectively, are both 0.5 s^−1^. Except for [MAPK*_A_*]*_total_* and 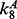, all other total molecular concentrations and reaction rates are kept fixed at the values given in Table S1. The broken lines indicate the physiologically plausible range of values for [MAPK*_A_*]*_total_* in (A) and 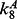 in (B), as used in the Huang-Ferrell model [45].

**Figure 5:**
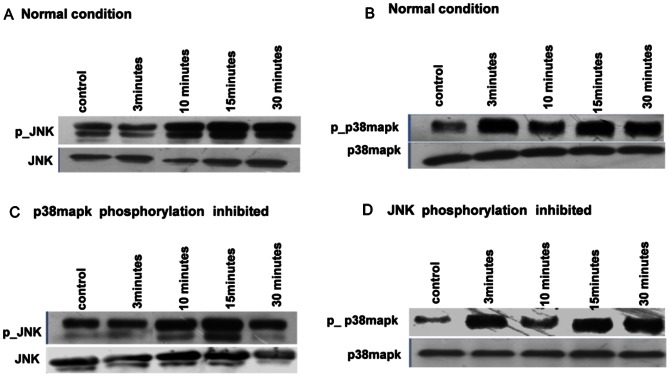
Experimental validation of the amplification of activity in one branch on inhibiting the activity of the terminal kinase in the other branch. Western blots of JNK and p38MAPK phosphorylation in primary macrophages when subjected to CD40 stimuli of strength 3 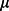
g/ml are shown under normal (JNK: A, p38MAPK: B) and perturbed (JNK: C, p38MAPK: D) conditions. Perturbation is applied by inhibiting the phosphorylation of either p38MAPK (C) or JNK (D) by using pharmaceutical agents. In each figure, the upper and lower panels show the phosphorylated and total concentrations of the different molecular species (represented by “p JNK” and “JNK”, and “p- p38” and “p38” for JNK and p38MAPK respectively). The **control** condition shows an unstimulated system, while the times 3, 10, 15 and 30 minutes refer to observations after the system has been exposed to stimuli of the corresponding duration. All experiments have been carried out in triplicate and a representative set of blots are shown. The densitometric analysis of the blots is indicated in Table 1.

Although decrease in concentration of MAPK*_A_* (Fig. 3 A) and lowering the product formation rate in the reaction of MAPK*_A_* with MAP2K_A_** (Fig. 4 B) functionally serve the same purpose, viz., inhibiting the production of MAPK_A_**, the resulting effect on other kinases in the system as a result of retrograde information propagation are fundamentally different. While the increase (decrease) in [MAPK*_A_*] leads to increase (decrease) in [MAP2K_B_**] and [MAPK_B_**] respectively (Fig. 4 A), the exact opposite effect is observed in the B branch kinases when product formation rate is increased (decreased) (see Fig. 4 B). This reveals a reciprocal response machinery of the branched cascade when it is subjected to different inhibitor types with apparently similar function (i.e., inhibiting activation of MAPK*_A_*), viz., the siRNA-type inhibitor which inhibits a fraction of total concentration of MAPK*_A_* and the pharmaceutical inhibitor which blocks the ATP binding site of a fraction of total MAPK*_A_*. The simulations reveal that while concentration depletion of MAPK*_A_* results in depletion of [MAP2K_B_**] and [MAPK_B_**] (Fig. 4 A), suppressing the phosphorylation of MAPK*_A_* (Fig. 4 B) amplified the concentrations of the B branch kinases. Subsequently, we have conducted experiments on a two-branch cascade comprising JNK and p38MAPK molecules, observing their phosphorylation in unperturbed and perturbed conditions. The JNK and p38MAPK are the terminal MAPK molecules belonging to two distinct pathways which share a common branch-point MAP3K molecule MEKK3/4 [19], [20]. In the experiments, stimulation of CD40 receptor (that acts as input to both JNK and p38MAPK pathways [23]) in macrophage cells from BALB/c mice by a ligand dose leads to JNK (Fig. 5 A) and p38MAPK phosphorylation (Fig. 5 B) in comparable magnitudes. The system is next subjected to ATP blockers that inhibit phosphorylation of either p38MAPK (Fig. 5 C) or JNK (Fig. 5 D). The densitometric analysis of the western blots (Figs. 5 A–D) in Table 1 shows the relative increase in phosphorylation of MAPK for the perturbed and unperturbed conditions.

**Table 1:** Densitometric analysis of western blots for JNK and p38MAPK under normal and perturbed conditions at different time points.

	Normal Condition						p38MAPK phosphorylation inhibited			JNK phosphorylation inhibited		
	[JNK]*_total_*	[JNK*] +[JNK**]	r_JNK_	[p38]_total_	[p38*] +[p38**]	r_p38_	[JNK]*_total_*	[JNK*] +[JNK**]	r*_JNK_*	[p38]*_total_*	[p38*] +[p38**]	r_p38_
Control	1	1	1	1	1	1	1	1	1	1	1	1
3 min	0.79	1.2	0.66	1.92	1.05	1.84	1.25	0.87	1.43	4.11	0.87	4.7
10 min	1.44	0.74	1.95	1.46	1.07	1.36	1.64	1.26	1.3	2.27	0.97	2.34
15 min	1.62	1.28	1.27	1.84	0.98	1.89	2.08	1.17	1.78	4.12	1.24	3.34
30 min	1.52	1.51	1.01	1.71	0.9	1.91	1.12	0.71	1.57	4.56	1.05	4.34

The observations show the change in activity as a function of exposure to stimuli applied for different durations. The perturbations correspond to inhibiting p38MAPK phosphorylation by a pharmaceutical agent followed by measurement of JNK activity, and conversely, inhibiting JNK phosphorylation by a pharmaceutical agent followed by measurement of p38 activity. The column headings [JNK]*_total_* and [p38]*_total_* refer to the total concentrations of the respective kinase molecules, [JNK*]+[JNK**] and [p38*]+[p38**] correspond to the concentrations of the respective (single or double) phosphorylated forms, while r*_JNK_* and r_p38_ represent the ratio of phosphorylated kinase to total kinase concentrations. *Control* refers to the unstimulated system, while the different times (viz., 3 minutes, 10 minutes, 15 minutes and 30 minutes) correspond to the duration for which the stimulus is applied.

The experiments validates the key prediction from our model: blocking the phosphorylation of MAPK*_A_* by targeting the ATP binding site enhances the phosphorylation of MAPK*_B_* (Table 1). As there is no experimental evidence of explicit feedback regulation between JNK and p38MAPK, it is extremely likely that the observed modulation of phosphorylation amplitudes of JNK and p38MAPK emerges as a result of long-range coordination through retrograde information propagation. The experiments additionally show that under perturbation, the retrograde propagation from JNK to p38MAPK is significantly higher than from p38MAPK to JNK (Table 1). Plausible reasons for this could lie in the asymmetry of the molecular concentration values and/or the reaction parameters in the two branches. Thus, we next analyze the effect of such asymmetry on the retrograde propagation of information.

**Figure 6:**
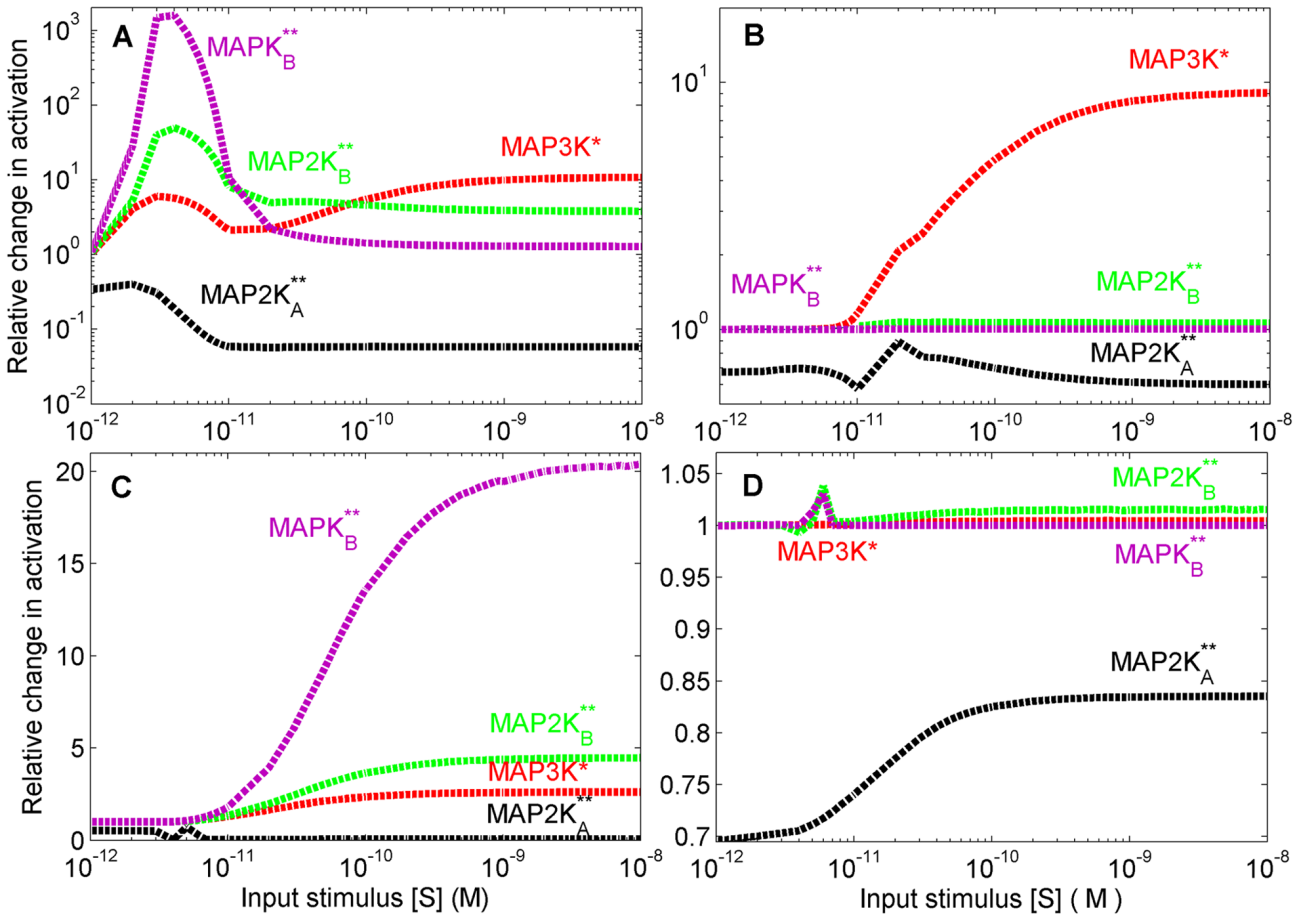
Role of branch asymmetry. Relative increase in response as a function of signal concentration on blocking MAPK*_A_* activation when the binding reaction rates for (A) branch A (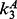, 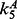 and 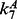) or (B) branch B (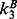, 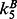 and 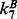) are 10 times higher than those in the other branch, and, when the total concentrations of MAPK and MAP2K in (C) branch A or (D) branch B are 10 times higher than the corresponding values for the other branch ( = mean value in the Huang-Ferrell range). Note that in (A) the MAPK*_B_* activation can increase by more than 1000 times for a particular range of signal strength. In contrast, there is relatively little change in the activity of the two branches when the activation of the terminal kinase for the branch having lower values of reaction rates is blocked.

We observe that when the ESK complex binding rates (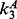, 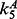 and 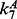) in the perturbed branch A are much higher (viz., by a factor of 10) than those in the unperturbed branch B (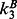, 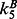 and 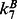), there is a remarkable increase in the activity of the latter branch, e.g., by three orders of magnitude in [MAPK_B_**], over a certain range of signal strength S (Fig. 6 A) as compared to the opposite situation, i.e., when the binding rates of the unperturbed branch are much higher than those of the perturbed branch (Fig. 6 B; see also Fig. S1). Also, when the total concentrations of MAPK and MAP2K in the perturbed branch A are much larger (viz., by a factor of 10) than the corresponding quantities in the unperturbed branch B, on blocking activation of MAPK*_A_* there is an observable increase in the activity of the unperturbed branch, e.g., about 20-fold increase in [MAPK_B_**] over a range of input stimuli strength (Fig. 6 C), compared to the case when the total concentrations of MAPK and MAP2K in the unperturbed branch are higher than the perturbed branch (Fig. 6 D). These results indicate that the extent of retrograde information propagation seen in a signaling cascade will depend on the magnitude of forward reaction flux (i.e., the strength of the flow “down” the cascade from MAP3K to MAPK) in the different branches of the system. If the branch having a larger downstream flux (resulting from relatively higher binding rates or larger total concentrations of the molecules belonging to that branch) is perturbed by inhibiting the activation of its terminal kinase, this will result in a much larger proportional increase in access to MAP3K for molecules in the unperturbed branch. On the other hand, if the branch having a lower magnitude of forward reaction flux is perturbed, the resulting increase in the activation of the unperturbed branch will be marginal as compared to the already higher activation levels of this branch in the control condition. This is further corroborated by the effect of asymmetry in other parameters of the perturbed and unperturbed branches, viz., (i) the product formation rates in the activation enzyme-substrate reactions, (ii) the binding rates with phosphatases in the deactivation reactions and (iii) the total phosphatase concentrations (Fig. S2). Thus, the experimentally observed asymmetry in the response of JNK and p38MAPK (Fig. 5) can be explained as a result of the differences in the reaction parameters or total concentrations of components of the two pathways.

**Figure 7:**
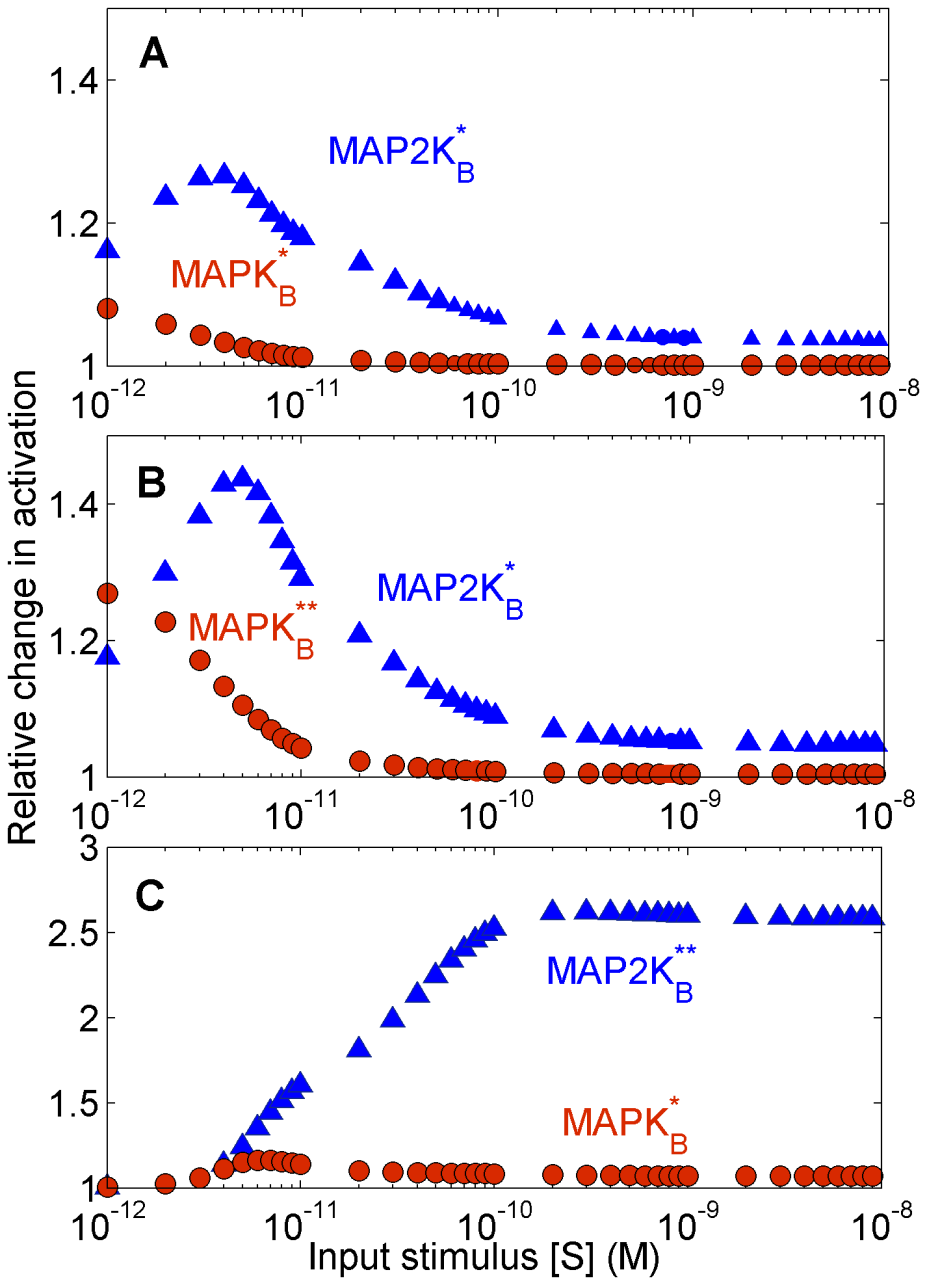
Role of competitive inhibition. Relative increase with stimulus strength of the steady-state response of different molecular species in the unperturbed branch B (MAP2K*_B_* and MAPK*_B_*) shown as a function of signal concentration, when phosphorylation of MAPK*_A_* is prevented. In (A) both MAP2K and MAPK are *singly* phosphorylated, while in (B) MAP2K are *singly* phosphorylated but MAPK are *doubly* phosphorylated. Note that there is a small increase in the steady-state response of MAP2K*_B_* and MAPK*_B_* in (B) compared to (A). (C) When MAP2K are *doubly* phosphorylated whereas MAPK are *singly* phosphorylated, the relative increase in steady-state activity of MAP2K*_B_* and MAPK*_B_* is more prominent.

Previous demonstrations of implicit feedback in linear signaling pathways had identified sequestration as the key mechanism [14], [24]. However, in the complex branched network motif investigated here, there are additional effects contributing to the retrograde propagation of information. In particular, we note the presence of competitive inhibition, e.g., through competition between singly phosphorylated and unphosphorylated forms of a substrate molecule (e.g., MAPK_A_* and MAPK*_A_*) for the common kinase enzyme (e.g., MAP2K_A_**). We have investigated the contribution of such competition in producing retrograde propagation by comparing the system with an artificial cascade model that allows only single phosphorylation of MAPK*_A,B_* and MAP2K*_A,B_* so that competitive inhibition is absent. We observe that the magnitude of retrograde propagation (and hence the amplification of kinase activation in the unperturbed branch) for the system with singly phosphorylated MAP2K and MAPK is significantly reduced (Fig. 7 A) compared to the original branched motif where the corresponding kinase molecules are doubly phosphorylated (Fig. 3 A). Retrograde propagation is also perceptibly weaker in model systems with reduced competitive inhibition, viz., where either (i) only MAP2K is singly phosphorylated while MAPK is doubly phosphorylated (Fig. 7 B), or (ii) only MAPK is singly phosphorylated while MAP2K is doubly phosphorylated (Fig. 7 C); however both exhibit higher degree of amplification of kinase activity in the unperturbed branch as compared to the situation when there is no competitive inhibition (Fig. 7 A).

**Figure 8:**
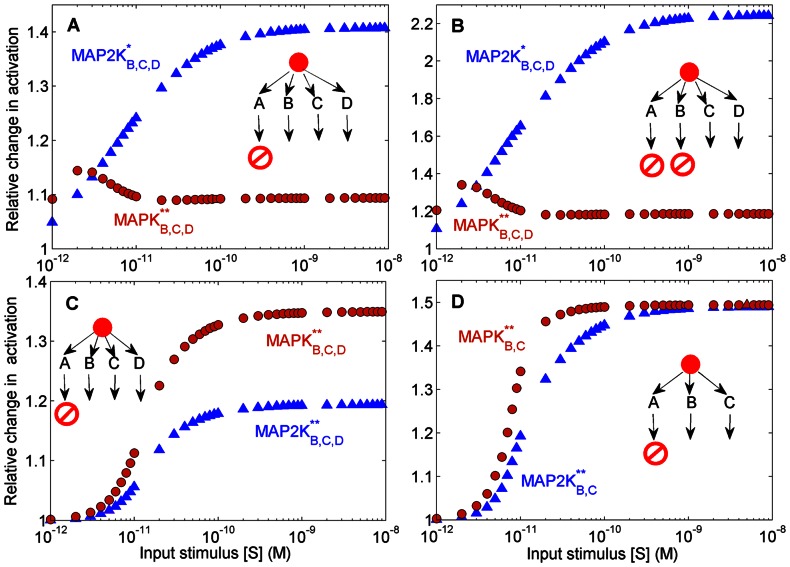
Effect of multiple branches. (A–B) Relative increase in steady-state response in a *four*-branch cascade which allows only *single* phosphorylation of MAP2K while MAPK is *doubly* phosphorylated as a function of stimulus strength for the cases (A) when MAPK*_A_* phosphorylation is prevented and (B) when phosphorylation of in two branches, i.e., of both MAPK*_A_* and MAPK*_A_*, are blocked. The effect of having four branches but with singly phosphorylated MAP2K is similar to having two branches with double phosphorylated MAP2K but with a lower relative change in the response. This suggests that competitive inhibition is playing a role but is not solely responsible for the retrograde propagation in the branched cascade model. (C–D) Relative increase in steady-state response of *doubly* phosphorylated MAP2K and MAPK in the unperturbed branches as a function of signal strength when phosphorylation of MAPK*_A_* is prevented in a (C) a *four*-branch and (D) a *three*-branch cascade. As the number of branches are increased, the relative change in MAP2K activity on perturbation decreases faster than that of MAPK.

Thus, sequestration effects inherent to the cascade reaction mechanism, as well as, competition between multiple substrates (e.g., MAP2K*_A_*, MAP2K*_B_*, MAP2K_A_* and MAP2K_B_*) for the same enzyme (MAP3K*), both contribute to the magnitude of retrograde propagation seen in a branched network. If these are the only factors affecting the degree of amplification of kinase activity in the unperturbed branch then the results of the original two-branch system should be reproducible in a hypothetical four-branch model network with doubly phosphorylated MAPK but only *singly* phosphorylated MAP2K (Figs. 8 A-B). When a single branch in such a system is perturbed (e.g., by inhibiting the phosphorylation of MAPK*_A_*), the effect on the unperturbed branches is relatively less compared to the original system where the MAP2K molecules are doubly phosphorylated (Fig. 3 A). In the former situation, the four competing substrates (MAP2K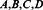) are all present initially, whereas, in the original network model (as well as in the experimental system comprising JNK and p38MAPK pathways), two of the competing substrates (MAP2K*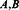) are the reaction products of the other two competing substrates (MAP2K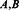) and are not present initially. Thus, in the latter case, the rise in concentrations of (and hence, the resulting competition from) two of the competing substrates has a time-delay with respect to the remaining two, making this system fundamentally different from the four-branch cascade. Further, double phosphorylation of MAP2K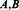 introduces an additional delay in the activation dynamics of the downstream MAPKs.

Based on these results, the extent of retrograde propagation of information in a branched network structure is seen to depend on multiple factors, viz.,: (i) the competition between branches for a common enzyme at the branch-point, (ii) sequestration of a kinase through binding in an ESK complex [24] and (iii) competitive inhibition between the un-phosphorylated and singly phosphorylated forms of the same kinase (capable of double phosphorylation) for its enzyme [25]. Fig. 8 (B) shows the relative increase of response in a four-branch network where the MAP2K molecules are phosphorylated only at a single site while the MAPK molecules are doubly phosphorylated, when the activation of MAPK in two branches (A and B) is prevented. For a range of strengths of the input stimulus, it is seen that the retrograde flow of information on blocking activation in two branches is higher as compared to blocking only one branch (compare Figs. 8 A and B). On the other hand, we observe that allowing double phosphorylation of MAP2K molecules (Fig. 8 C) results in a stronger response (compared to allowing only single phosphorylation of MAP2K as in Fig. 8 A) in the unperturbed branches (B,C,D) when activation of MAPK in one branch (A) is blocked. Note that in a four-branch cascade with double phosphorylation of MAPK and MAP2K, the relative increase in activity resulting from the perturbation is higher for MAPK as compared to MAP2K in the unperturbed branches (which is opposite to the situation observed in a two-branch network). However, when the number of branches is reduced from four to three (Fig. 8 D) the relative increase of activity for MAPK and MAP2K in the unperturbed branches (B,C) become comparable. Thus, comparing Figs. 8 (C–D) and 3 (A), we note that as the number of branches increase, on inhibiting the activation of the terminal molecule (MAPK) in one of the branches, the resulting relative increase in activity of the MAP2K molecules in the unperturbed branches is reduced, while that of the corresponding MAPK molecules remains comparatively unchanged.

In a biological setting, the concentrations of kinases and phosphatases are usually of comparable magnitude and the systems are exposed to a wide range of signal strengths [24], [26]; our results are valid under these realistic conditions. We also stress that our results have been obtained by simulating the full dynamical model without using quasi-steady-state assumptions that focus exclusively on steady-state behavior. Such approximations ignore rapid transient changes in the concentrations of signaling molecules and do not reproduce the effect of feedback interactions [27], [28]. We also note that critical biological properties should be robust against parameter changes [29], caused by variations in the environment, polymorphisms or mutations [30] that can influence not only a single parameter but many of them simultaneously [13]. Thus, if retrograde propagation of information is indeed expected to play a significant role in intracellular signaling it should be robust. We have established the robustness of our observations by verifying that the results are not sensitively dependent on system parameters (Fig. S3).

## Discussion

Our demonstration that local intervention in a signaling network can have remarkable non-local consequences has implications for understanding how the intricate machinery of information processing functions in the cell. In particular, reciprocal inhibition between parallel pathways can occur without the involved agents directly reacting with each other. This allows a high level of adaptability in control without a concomitant increase in the wiring complexity in the network, leading to a more efficient system design. Long-range effects also assume importance in light of the current experimental paradigm in systems biology where the observation of up- or down-regulation of activity for a molecule as a result of perturbing another molecule is assumed to indicate the existence of a direct interaction between them [31]. While interconnections in a signaling network are often inferred on the basis of such observations, our results show that dynamical correlations between molecular activities may have a fundamentally different origin. Note that although we have used the MAPK module to illustrate the mechanism of retrograde propagation, it is conceivable that branching in the signaling network can occur upstream of MAP3K resulting in indirect communication over even larger distances in the system.

The results reported here have potential significance for designing drugs against systems-level diseases such as cancer that proliferate through complex orchestration of multiple signaling molecules [32]–[34]. Such diseases are multi-factorial and may have multiple possible targets for pharmaceutical intervention. Conversely, many drugs that are used for treatment may be working through as yet undetermined mechanisms, so that even if they have a known target, there can be potentially undesirable ‘off-target’ effects [35]. From the point of view of drug design for systems-level diseases, the crucial implication of the results reported here is that the effect of competitive blocking of a ATP binding site with a pharmaceutical agent may be completely different from the effect of suppressing the kinase expression by siRNA mediated inhibition. This is critical when one considers the ‘off-target’ effect of such intervention on the activity of other molecules in the system which may also be playing a crucial role in the disease. In fact, these two methods are classically assumed to have the same effect on the system and siRNA experiments are often used to validate the model driven hypothesis suggested by experiments involving pharmaceutical inhibitors. Thus, the physiological impact of retrograde information propagation is realized with dramatic effect in branched signaling cascades as the multiple pathways show strikingly different response to apparently similar interventions.

We can, for example, consider the proteins JNK and p38MAPK, both of which are now established to be intimately involved in the proliferation of cancer and its cure. However, the respective functions of these two molecules in cancer development are not well-understood and their contribution to genesis and propagation of cancer may sometimes appear to be contradictory [36]. Certain cells use these signalling pathways to oppose cell proliferation and morphological transformation, whereas cancer cells can subvert these pathways to facilitate proliferation, survival and invasion. For example, while the JNK and p38MAPK can both act as pro-apoptotic pathways that may help cure cancer, they have also been found to function as oncoproteins that help cancer cells survive [36]. Hence depending on the cellular context, the JNK and p38MAPK pathways could be used by the cell either to deliver complementary outputs, or, to trigger antagonistic cells fates [36]. Under these circumstances, drug design for such diseases should take into consideration the system-level consequence of inhibiting a particular kinase on the activity of other kinases. This can have consequences for drug therapy as one can control the activity of a molecular species with a pharmaceutical agent that interacts with a different molecule, provided the two species are indirectly related by the long-range control mechanism described here.

Complexity of a signaling network [10], [11] may be increased further through inter-modular cross-talk [12]. In principle, there can be multiple inputs impinging on a signaling motif, as well as, interference between signals traveling through different pathways activated by the same receptor. For example, it is observed that the kinase Cot can activate the ERK MAPK independent of the corresponding MAP3K (Raf) [37]. This implies that there can be additional inputs to MAP2K, apart from its usual MAP3K. For such a situation in the branched module discussed here, retrograde propagation will affect all the inputs, its magnitude varying according to the strength of crosstalk between the additional input and the branched MAPK cascade. Thus, the perturbation of a terminal kinase will not only affect the immediate module of which it is part, but through the interaction of the module with other inputs the retrograde propagation of information can connect apparently remote and unrelated modules of the network, e.g., through Cot the perturbation of MAPK may eventually affect regulation of IkB kinase [38].

We have shown here that different types of perturbations having the same objective (e.g., both siRNA inhibitor as well as ATP binding blocker aims at reducing MAPK activity), while having similar consequences in a linear signaling cascade, can give rise to very different results in a branched network. Such distinct responses to apparently similar perturbations may be crucial when dealing with co-regulated diseases. For example, JNK signaling is enhanced and p38MAPK signaling is abrogated in different cancers [36], [39]. On the other hand, both JNK and p38MAPK signaling promotes diabetes by negatively regulating the function of insulin receptor [40], [41]. Co-diagnosis of both cancer and diabetes in the same individual is not uncommon, although the system level causality behind such occurrence is not understood [42]. More importantly, the protein IRS1 (insulin receptor substrate 1) which has a critical role in insulin-signaling pathways and whose mutation is known to result in genetic risk for type 2 diabetes [43], has been identified as a drug target for cancer [44]. We can qualitatively argue from our analysis that pharmaceutical intervention designed to inhibit JNK phosphorylation for suppressing cancer might result in prolonged enhancement of p38MAPK signaling which could consequently exceed a cellular threshold, worsening any pre-existing diabetic condition. Thus, while an ATP blocker-type drug can be used to inhibit JNK phosphorylation, if the resulting increase in p38MAPK phosphorylation is undesirable, a better option is to use an inhibitor reducing JNK expression. Such insights on the differential effects of drugs designed for the same disease is an important outcome of studying how the local dynamical properties of modules (such as the branched cascade motif) can affect the function of the larger signaling networks in which they are embedded [28].

Our results may also be used to understand the evolutionary advantage of intracellular pathogens which only target molecules in one branch of parallel MAPK pathways but which can nevertheless modulate the cellular response to its own advantage. These pathogens often target the MAPK module as the upstream signaling intermediates converge to MAPK for final integration of the signal deciding the cellular responses. As a result, the pathogens do not need to devise extremely complicated interception strategies targeting many types of molecules in order to survive within the host. As the signal strength cannot be adjusted beyond the MAPK module, the pathogen strategy would be a winning one. The retrograde propagation capability of a branched motif described here can explain how host cell signaling can still be adjusted to maintain the MAPK-dependent cellular functions.

## Materials and Methods

### Mathematical Modeling

The time-evolution of the different molecular concentrations in the branched motif has been described using a set of coupled ordinary differential equations (ODEs) where each enzyme (E)-substrate kinase (SK) reaction has two steps: (i) a reversible enzyme-substrate kinase complex (ESK) formation step (with the forward and reverse reaction rates denoted by 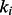 and 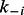 for the 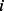-th reaction) and (ii) an irreversible step of product (i.e., the activated substrate SK*) formation from the complex (with a rate 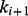). In a linear cascade, the product (activated substrate) of an earlier step can be the enzyme for the next step; thus, a MAP3K-MAP2K-MAPK reaction cascade (as in the Huang-Ferrell model [45], considering the kinase as well as phosphatase-mediated reactions) is described by 18 coupled ODEs while our model with two MAP2K-MAPK branches emerging from a common MAP3K is described by 32 coupled ODEs. For simplicity, the branches have been considered to be symmetric and the corresponding parameters in each branch are assigned the same values. The system of coupled ODEs are solved numerically using the ode23s routine for solving stiff equations implemented in Matlab 7, without invoking the quasi-steady-state hypothesis of Michelis-Menten kinetics [27]. The model parameters, viz., the different reaction rates and initial concentrations of the substrate molecules, are adapted from the Huang-Ferrell model [13], [45] (see Table S1).

### Analysis of Robustness with Respect to Parameter Variation

Robustness analysis for the results reported here with respect to variation in the parameters have been carried out by performing Monte Carlo simulations over 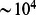 realizations of the model system. In each realization, the 38 model parameters are randomly sampled from a biologically plausible range [13], [45] having an uniform distribution about the respective Huang-Ferrell (HF) values (Table S1). The branches have been considered to be symmetric with corresponding parameters in each branch assigned identical values. The relative increase in the activity of MAP2K_B_** and MAPK_B_** on blocking MAPK*_A_* activation are measured at five different signal concentrations between [S] = 10^−12^M and 10^−8^M (a total of 65500 random realizations). The robustness of the system response is measured as a function of the degree of variation in its parameter values quantified by the Total Parameter Variation (TPV) [29]:
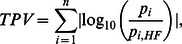
where p*_i_* denotes the value of the i*^th^* parameter in a given realization and p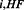 is the reference value of the parameter as given in the Huang-Ferrell model (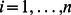). Fig. S3 shows the relative increase in steady state activity of MAPK_A_** and MAP2K_B_** on blocking MAPK*_A_* activation for signal strengths [S] = 10^−12^ M (A–B), 10^−10^ M (C–D), and 10^−8^ M (E–F) as a function of TPV. We observe that even relatively large deviations from the HF parameter values produce results qualitatively similar to that reported in our paper.

### Animals, Cell Lines and Parasite

BALB/c mice from Jackson Laboratories (Bar Harbor, ME) have been bred in our (NCCS) experimental facility. All animal experiments have been performed according to the animal use protocol approved by the Instiutional animal care and use committee [IAEC (Institutional Animal Ethics Committee) approved protocol number EAF-110; under the central control of the “Committee for the Purpose of Control and Supervision of Experiments on Animals (CPCSEA)” permit no. 7/1999/CPCSEA-09/03/1999]. P388D1, a macrophage-like cell line [46]–[49], is used for transfection studies. For infection, L. major strain (MHOM/Su73/5ASKH) has been used.

### Macrophage Lysate Preparation for Western Blot

Thioglycolate-elicited macrophages have been treated with an agonistic anti-CD40 antibody, as indicated in [46], followed by cell lysate preparation and Western blot for the kinases [46], [47], [49]. Antibodies have been procured from Cell Signaling Technology (Beverly, MA) and Santa Cruz Biotech (Santa Cruz, CA).

### Western Blot

In a 6-well tissue culture plate, 2×105 cells/well have been cultured in antibiotic-free RPMI-1640 supplemented with 10% FCS. The experiments have been performed three times and the data from a representative experiment has been shown.

### Inhibitor studies

Pharmaceutical inhibitors have been used for blocking the phosphorylation of JNK and p38MAPK. For blocking p38MAPK phosphorylation the agent SB203580 has been used and JNK phosphorylation has been blocked using SP600125. For the kinetic studies with inhibitors the cells have been pre-incubated with specific inhibitor type (SB203580 or SP600125) for 1 hour and subsequently the cells have been stimulated for the desired time.

### Quantization of the Signaling Intermediate Activation

Western blot data have been subjected to densitometry analysis and phosphorylation ratio (PR) = (Phosphorylated kinase/Total kinase) for individual kinase has been calculated. For the kinetics of phosphorylation in the wild-type condition and in kinase inhibition (p38MAPK and JNK), relative PR values at different time points under inhibited conditions have been compared to their respective controls. For the inhibitor studies PR before inhibition for all kinases has been scaled to 1 and the respective changes (increase or decrease) in the PR after inhibition have been calculated accordingly. To avoid possible artifacts resulting from transcription mediated feedback such as the induction of MAPK phosphatase, which can become significant at longer durations [48], the experiments are not continued beyond 30 minutes.

## Supporting Information

Figure S1
Role of competition between the two branches in binding to MAP3K*. Relative increase of response as a function of the ratio of the binding rates of MAP2K*_A_* (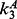 = 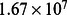 held constant) and MAP2K*_B_* (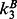) with MAP3K* on blocking (A) MAPK*_A_* activation and (B) MAPK*_B_* activation. Relative increase of response as a function of the ratio of the binding rates of MAP2K*_A_* (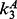) and MAP2K*_B_* (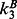 = 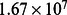 held constant) with MAP3K* on blocking (C) MAPK*_A_* activation and (D) MAPK***_A_*** activation.(TIF)

Figure S2
**Role of asymmetry for reaction parameters in the two branches.** Relative increase of response as a function of the signal on blocking MAPK*_A_* activation when (A) the product formation rates for branch A (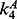 and 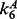) are 5 times higher than those in branch B and (B) the product formation rates for branch B (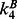 and 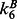) are 5 times higher than those in branch A; (C) the binding reaction rates for branch A (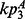 and 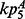) are 10 times higher than those in branch B; (D) the binding reaction rates for branch B (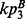 and 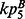) are 10 times higher than those in branch A; (E) the total concentrations of phosphatase of MAP2K* and MAP2K** of branch A are 10 times larger than the corresponding values for branch B ( = mean value in the Huang-Ferrell range) and (F) the total concentrations of phosphatase of MAP2K* and MAP2K** of branch B are 10 times larger than the corresponding values for branch A ( = mean value in the Huang-Ferrell range).(TIF)

Figure S3 **Robustness of the effect of retrograde propagation in branched motifs with respect to parameter variation.** The variation in the relative increase in response, i.e., concentrations of MAPK_B_** and MAP2K_B_** on blocking the phosphorylation of MAPK*_A_*, measured in terms of Total Parameter Variation (TPV) on randomly varying the 38 parameters in the model. The dots in each figure indicate the individual values obtained from 10^4^ realizations for three different signal strengths: (a–b) [S_0_] = 10^−12^ M, (c–d) [S_0_] = 10^−10^ M and (e–f) [S_0_] = 10^−8^ M. The 38 parameters, which include total concentrations and reaction rates for all kinases and phosphatases at a given branch (the corresponding values for the other branch are taken to be the same) are randomly chosen from uniform distributions bounded between physiologically plausible minimum and maximum values for the parameters that are given in Table S1.
(TIF)

Table S1 **Parameter values for reactant total concentrations and reaction rates used in the model simulation.** The same values for the corresponding parameters are used in the different branches.
(XLS)

Text S1 **Equations describing the dynamics of the model system with two branches.**
(PDF)
