# Supplementary figures and images for: Branched Motifs Enable Long-Range Interactions in Signaling Networks through Retrograde Propagation
Source: PLoS One. 2013 May 31;8(5):e64409. doi: 10.1371/journal.pone.0064409 (PMC3669326; doi:10.1371/journal.pone.0064409)

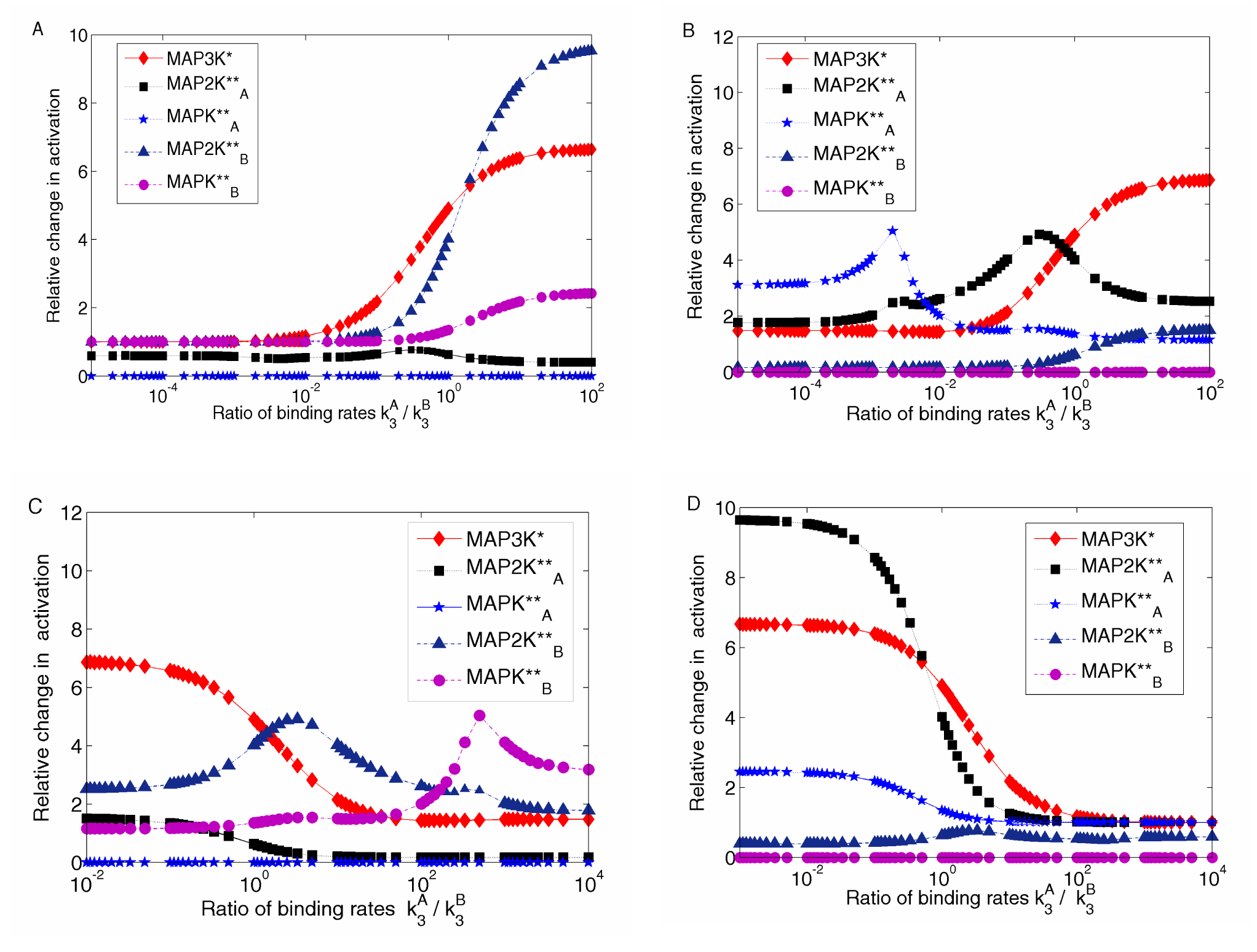

Supplement: Figure S1 — Role of competition between the two branches in binding to MAP3K*. Relative increase of response as a function of the ratio of the binding rates of MAP2KA ( = held constant) and MAP2KB () with MAP3K* on blocking (A) MAPKA activation and (B) MAPKB activation. Relative increase of response as a function of the ratio of the binding rates of MAP2KA () and MAP2KB ( = held constant) with MAP3K* on blocking (C) MAPKA activation and (D) MAPKA activation. (TIF) [file pone.0064409.s001.tif]

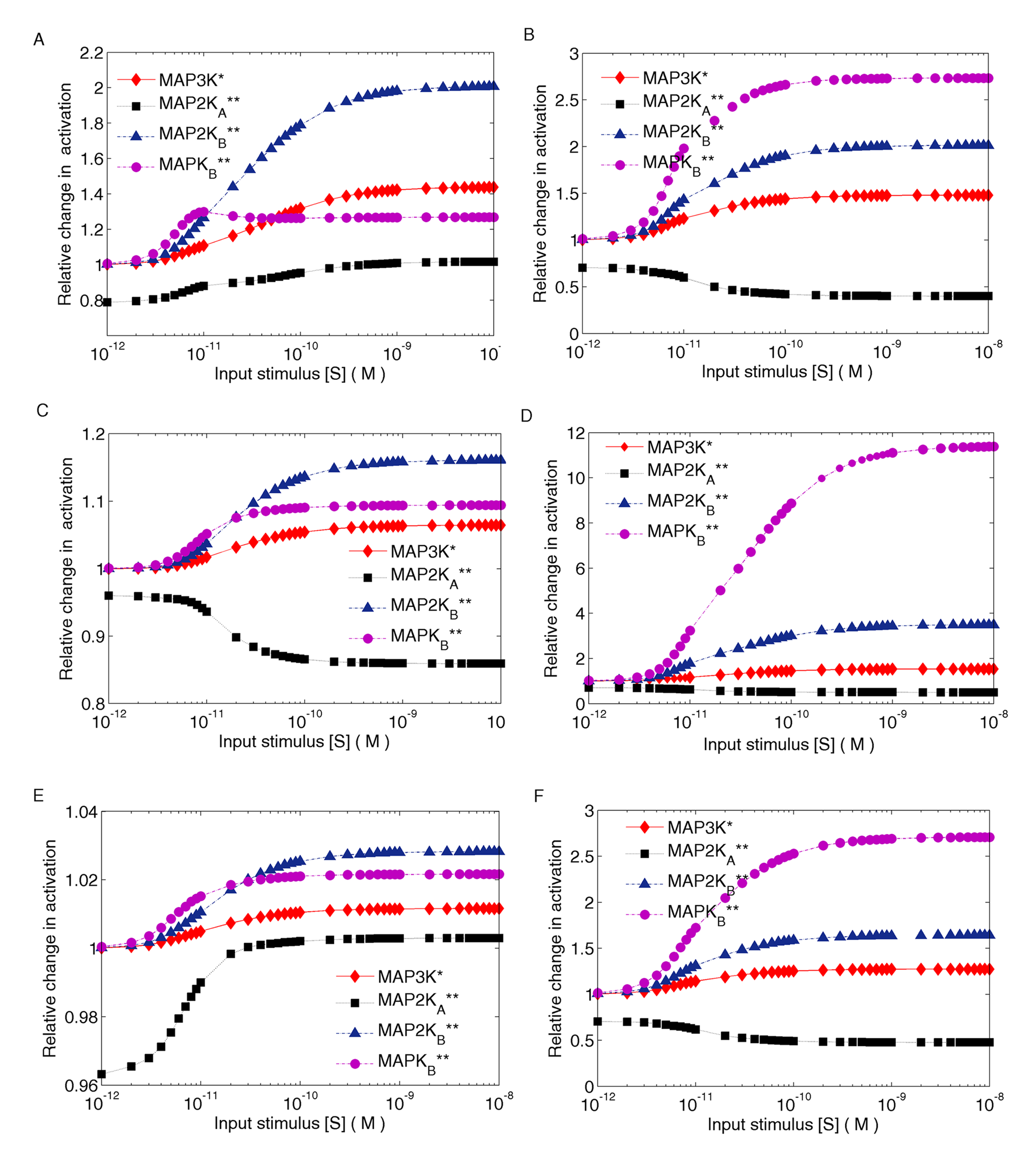

Supplement: Figure S2 — Role of asymmetry for reaction parameters in the two branches. Relative increase of response as a function of the signal on blocking MAPKA activation when (A) the product formation rates for branch A ( and ) are 5 times higher than those in branch B and (B) the product formation rates for branch B ( and ) are 5 times higher than those in branch A; (C) the binding reaction rates for branch A ( and ) are 10 times higher than those in branch B; (D) the binding reaction rates for branch B ( and ) are 10 times higher than those in branch A; (E) the total concentrations of phosphatase of MAP2K* and MAP2K** of branch A are 10 times larger than the corresponding values for branch B ( = mean value in the Huang-Ferrell range) and (F) the total concentrations of phosphatase of MAP2K* and MAP2K** of branch B are 10 times larger than the corresponding values for branch A ( = mean value in the Huang-Ferrell range). (TIF) [file pone.0064409.s002.tif]

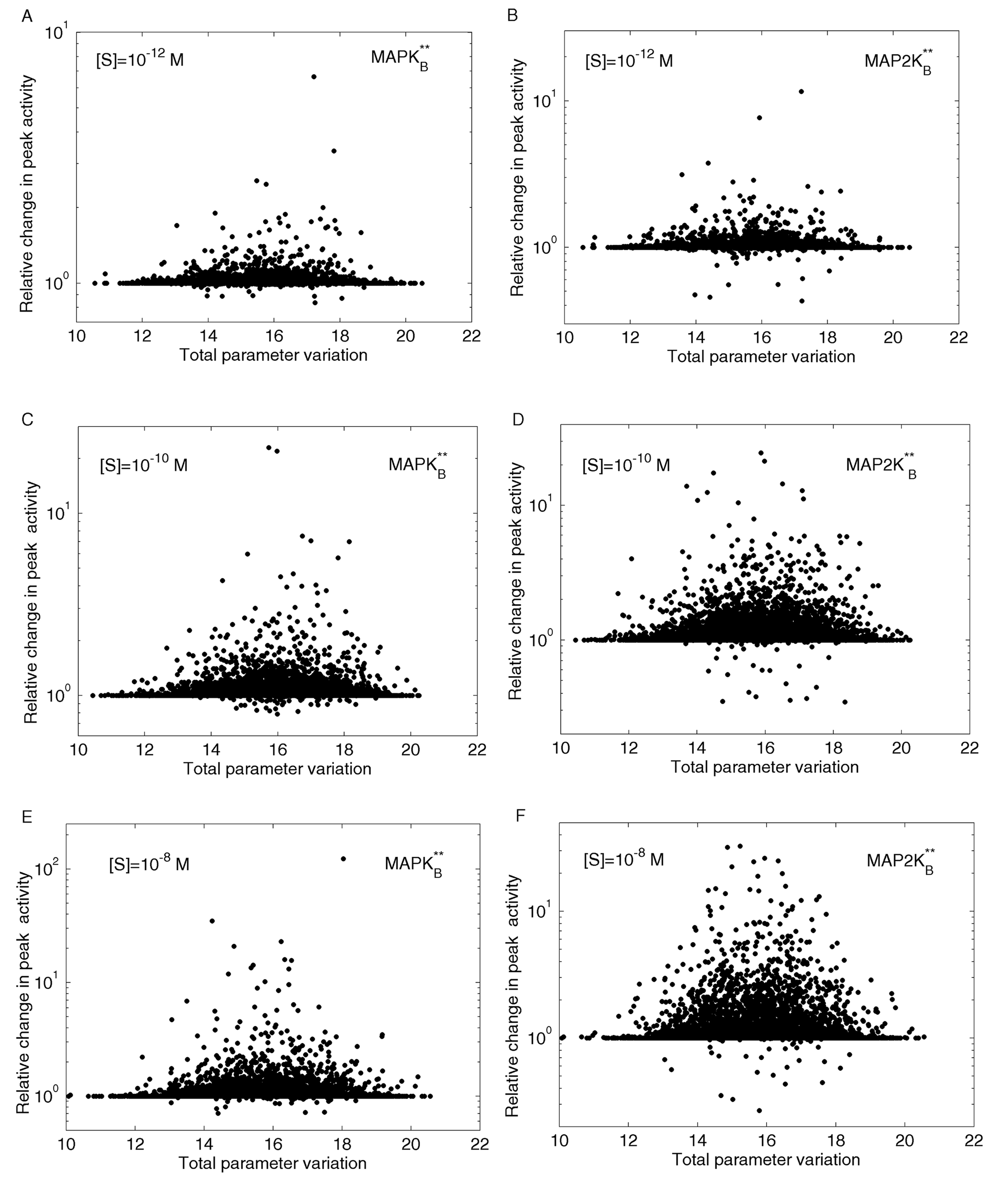

Supplement: Figure S3 — Robustness of the effect of retrograde propagation in branched motifs with respect to parameter variation. The variation in the relative increase in response, i.e., concentrations of MAPKB** and MAP2KB** on blocking the phosphorylation of MAPKA, measured in terms of Total Parameter Variation (TPV) on randomly varying the 38 parameters in the model. The dots in each figure indicate the individual values obtained from 104 realizations for three different signal strengths: (a–b) [S0] = 10−12 M, (c–d) [S0] = 10−10 M and (e–f) [S0] = 10−8 M. The 38 parameters, which include total concentrations and reaction rates for all kinases and phosphatases at a given branch (the corresponding values for the other branch are taken to be the same) are randomly chosen from uniform distributions bounded between physiologically plausible minimum and maximum values for the parameters that are given in Table S1. (TIF) [file pone.0064409.s003.tif]
