# Supplementary material for: Branched Motifs Enable Long-Range Interactions in Signaling Networks through Retrograde Propagation
Source: PLoS One. 2013 May 31;8(5):e64409. doi: 10.1371/journal.pone.0064409 (PMC3669326; doi:10.1371/journal.pone.0064409)
Supplement: Text S1 — Equations describing the dynamics of the model system with two branches. (PDF) [file pone.0064409.s005.pdf]

Text S1. Equations describing the Dynamics of the Model System with Two Branches.

The dynamics of the two-branched reaction cascade model (with common MAP3K) is described by the following enzyme–substrate reactions.

Branch point

Note: KKK denotes MAP3K and KKKP denotes MAP3K* Phosphatase

$$S + KKK \underset{k_{-1}}{\overset{k_1}{\rightleftarrows}} S.KKK \xrightarrow{k_2} S + KKK* \quad (1)$$

$$KKKP + KKK* \underset{kp_{-1}}{\overset{kp_1}{\rightleftarrows}} KKKP.KKK* \xrightarrow{kp_2} KKKP + KKK \quad (2)$$

Branch A

In the following equations KK_A denotes branch A $MAP2K_A$; KKP_A* denotes branch A $MAP2K_A$*Phosphatase; KKP_A** denotes branch A $MAP2K_A$**Phosphatase; K_A denotes branch A $MAPK_A$; KP_A* denotes branch A $MAPK_A$*Phosphatase; KP_A** denotes branch A $MAPK_A$**Phosphatase.

$$KKK*+KK_A \underset{k_{-3}^A}{\overset{k_3^A}{\rightleftarrows}} KKK*.KK_A \xrightarrow{k_4^A} KKK*+KK_A* \quad (3)$$

$$KKP_A*+KK_A* \underset{kp_{-3}^A}{\overset{kp_3^A}{\rightleftarrows}} KKP_A*.KK_A* \xrightarrow{kp_4^A} KKP_A*+KK_A \quad (4)$$

$$KKK*+KK_A* \underset{k_{-5}^A}{\overset{k_5^A}{\rightleftarrows}} KKK*.KK_A* \xrightarrow{k_6^A} KKK*+KK_A** \quad (5)$$

$$KKP_A**+KK_A** \underset{kp_{-5}^A}{\overset{kp_5^A}{\rightleftarrows}} KKP_A**.KK_A** \xrightarrow{kp_6^A} KKP_A**+KK_A* \quad (6)$$

$$KK_A**+K_A \underset{k_{-7}^A}{\overset{k_7^A}{\rightleftarrows}} KK_A**.K_A \xrightarrow{k_8^A} KK_A**+K_A* \quad (7)$$

$$KP_A*+K_A* \underset{kp_{-7}^A}{\overset{kp_7^A}{\rightleftarrows}} KP_A*.K_A* \xrightarrow{kp_8^A} KP_A*+K_A \quad (8)$$

$$KK_A**+K_A* \underset{k_{-9}^A}{\overset{k_9^A}{\rightleftarrows}} KK_A**.K_A* \xrightarrow{k_{10}^A} KK_A**+K_A** \quad (9)$$

$$KP_A**+K_A** \underset{kp_{-9}^A}{\overset{kp_9^A}{\rightleftarrows}} KK_A**.K_A** \xrightarrow{k_{10}^A} KP_A**+K_A* \quad (10)$$

Branch B

KK_B denotes branch B MAP2K_B; KKP_B* denotes branch B MAP2K_B*Phosphatase; KKP_B** denotes branch B MAP2K_B**Phosphatase; K_B denotes branch B MAPK_B; KP_B* denotes branch B MAPK_B*Phosphatase; KP_B** denotes branch B MAPK_B**Phosphatase.

$$KKK^* + KK_B \xrightleftharpoons[k_{-3}^B]{k_3^B} KKK^*.KK_B \xrightarrow{k_4^B} KKK^* + KK_B^* \tag{11}$$

$$KKP_B^* + KK_B^* \xrightleftharpoons[kp_{-3}^B]{kp_3^B} KKP_B^*.KK_B^* \xrightarrow{kp_4^B} KKP_B^* + KK_B \tag{12}$$

$$KKK^* + KK_B^* \xrightleftharpoons[k_{-5}^B]{k_5^B} KKK^*.KK_B^* \xrightarrow{k_6^B} KKK^* + KK_B^{**} \tag{13}$$

$$KKP_B^{**} + KK_B^{**} \xrightleftharpoons[kp_{-5}^B]{kp_5^B} KKP_B^{**}.KK_B^{**} \xrightarrow{kp_6^B} KKP_B^{**} + KK_B^* \tag{14}$$

$$KK_B^{**} + K_B \xrightleftharpoons[k_{-7}^B]{k_7^B} KK_B^{**}.K_B \xrightarrow{k_8^B} KK_B^{**} + K_B^* \tag{15}$$

$$KP_B^* + K_B^* \xrightleftharpoons[kp_{-7}^B]{kp_7^B} KP_B^*.K_B^* \xrightarrow{kp_8^B} KP_B^* + K_B \tag{16}$$

$$KK_B^{**} + K_B^* \xrightleftharpoons[k_{-9}^B]{k_9^B} KK_B^{**}.K_B^* \xrightarrow{k_{10}^B} KK_B^{**} + K_B^{**} \tag{17}$$

$$KP_B^{**} + K_B^{**} \xrightleftharpoons[kp_{-9}^B]{kp_9^B} KK_B^{**}.K_B^{**} \xrightarrow{k_{10}^B} KP_B^{**} + K_B^* \tag{18}$$

The 18 enzyme-substrate reactions given above are described by 32 ordinary differential equations (ODEs) in the model.

$$\frac{d[KKK]}{dt} = k_{-1}.[S.KKK] + kp_2.[KKKP.KKK^*] - k_1.[S].[KKK] \tag{1}$$

$$\frac{d[S.KKK]}{dt} = k_1.[S].[KKK] - (k_{-1} + k_2).[S.KKK] \tag{2}$$

$$\frac{d[KKKP.KKK*]}{dt} = kp_1.[KKKP].[KKK*] - (kp_2 + kp_{-1}).[KKKP.KKK*] \tag{3}$$

$$\begin{aligned}\frac{d[KKK*]}{dt} = {} & k_{2.}.[S.KKK] + kp_{-1}[KKKP.KKK*] - kp_1.[KKKP].[KKK*] \\ & + (k_{-3}{}^{A} + k_4{}^{A}).[KKK*.KK_A] - k_3{}^{A}[KKK*].[KK_A] \\ & + (k_{-5}{}^{A} + k_6{}^{A}).[KKK*.KK_A*] - k_5{}^{A}.[KKK*].[KK_A*] \\ & + (k_{-3}{}^{B} + k_4{}^{B}).[KKK*.KK_B] - k_3{}^{B}[KKK*].[KK_B] \\ & + (k_{-5}{}^{B} + k_6{}^{B}).[KKK*.KK_B*] - k_5{}^{B}.[KKK*].[KK_B*]\end{aligned} \tag{4}$$

$$\frac{d[KK_A]}{dt} = k_{-3}{}^{A}.[KKK*.KK_A] + kp_4{}^{A}.[KKP_A*.KK_A*] - k_3{}^{A}.[KKK*].[KK_A] \tag{5}$$

$$\frac{d[KKK*.KK_A]}{dt} = k_3{}^{A}[KKK*].[KK_A] - (k_{-3}{}^{A} + k_4{}^{A}).[KKK*.KK_A] \tag{6}$$

$$\frac{d[KKP_A*.KK_A*]}{dt} = kp_3{}^{A}[KKP_A*].[KK_A*] - (kp_4{}^{A} + kp_{-3}{}^{A}).[KKP_A*.KK_A*] \tag{7}$$

$$\begin{aligned}\frac{d[KK_A*]}{dt} = {} & kp_5{}^{A}.[KKK*KK_A] + kp_{-3}{}^{A}.[KKP_A*.KK_A*] - kp_3{}^{A}.[KKP_A*].[KK_A*] \\ & + k_{-5}{}^{A}.[KKK*.KK_A*] - k_5{}^{A}.[KKK*].[KK_A*] + kp_6{}^{A}.[KKP_A**.KK_A**]\end{aligned} \tag{8}$$

$$\frac{d[KKK*.KK_A*]}{dt} = k_5{}^{A}.[KKK*].[KK_A*] - (k_6{}^{A} + k_{-5}{}^{A}).[KKK*.KK_A] \tag{9}$$

$$\frac{d[KKP_A**.KK_A**]}{dt} = kp_5{}^{A}.[KKP_A**].[KK_A**] - (kp_6{}^{A} + kp_{-5}{}^{A}).[KKP_A**.KK_A**] \tag{10}$$

$$\frac{d[KK_A **]}{dt} = k_6{}^A.[KKK*.KK_A*] + kp_{-5}{}^A.[KKP_A**.KK_A**] - kp_5{}^A.[KKP_A**].[KK_A**]$$

$$+ (k_{-7}{}^A + k_8{}^A).[KK_A**.K_A] - k_7{}^A.[KK_A**].[K_A]$$

$$+ (k_{-9}{}^A + k_{10}{}^A).[KK_A**.K_A*] - k_9{}^A.[KK_A**].[K_A*] \qquad (11)$$

$$\frac{d[K_A]}{dt} = k_{-7}{}^A.[KK_A**.K_A] + kp_8{}^A[KP_A*.K_A*] - k_7{}^A.[KK_A**].[K_A] \qquad (12)$$

$$\frac{d[KK_A**.K_A]}{dt} = k_7{}^A.[KK_A**].[K_A] - (k_8{}^A + k_{-7}{}^A).[KK_A**.K_A] \qquad (13)$$

$$\frac{d[KP_A*.K_A*]}{dt} = kp_7{}^A.[KP_A*].[K_A*] - (kp_{-7}{}^A + kp_8{}^A).[KP_A*.K_A*] \qquad (14)$$

$$\frac{d[K_A*]}{dt} = k_8{}^A.[KK_A*.K_A] + kp_{-7}{}^A.[KP_A*.K_A*] - kp_7{}^A.[KP_A*].[K_A*]$$

$$+ k_{-9}{}^A.[KK_A**.K_A*] - k_9{}^A.[KK_A**].[K_A*] + kp_{10}{}^A[KP_A**.K_A**] \qquad (15)$$

$$\frac{d[KK_A**.KA*]}{dt} = k_9{}^A.[KK_A**].[K_A*] - (k_{-9}{}^A + k_{10}{}^A).[KK_A**.K_A*] \qquad (16)$$

$$\frac{d[KP_A**.K_A**]}{dt} = kp_9{}^A.[KP_A**].[K_A**] - (kp_{-9}{}^A + kp_{10}{}^A).[KP_A**.K_A**] \qquad (17)$$

$$\frac{d[K_A**]}{dt} = k_{10}{}^A.[KK_A**.K_A*] + kp_{-9}{}^A.[KP_A**.K_A**] - kp_9{}^A.[KP_A**].[K_A**] \qquad (18)$$

$$\frac{d[KK_B]}{dt} = k_{-3}{}^B.[KKK*.KK_B] + kp_4{}^B.[KKP_B*.KK_B*] - k_3{}^B.[KKK*].[KK_B] \qquad (19)$$

$$\frac{d[KKK*.KK_B]}{dt} = k_3{}^B[KKK*].[KK_B] - (k_{-3}{}^B + k_4{}^B).[KKK*.KK_B] \quad (20)$$

$$\frac{d[KKP_B*.KK_B*]}{dt} = kp_3{}^B[KKP_B*].[KK_B*] - (kp_4{}^B + kp_{-3}{}^B).[KKP_B*.KK_B*] \quad (21)$$

$$\frac{d[KK_B*]}{dt} = kp_5{}^B.[KKK*KK_B] + kp_{-3}{}^B.[KKP_B*.KK_B*] - kp_3{}^B.[KKP_B*].[KK_B*]$$

$$+ k_{-5}{}^B.[KKK*.KK_B*] - k_5{}^B.[KKK*].[KK_B*]$$

$$+ kp_6{}^B.[KKP_B**.KK_B**] \quad (22)$$

$$\frac{d[KKK*.KK_B*]}{dt} = k_5{}^B.[KKK*].[KK_B*] - (k_6{}^B + k_{-5}{}^B).[KKK*.KK_B] \quad (23)$$

$$\frac{d[KKP_B**.KK_B**]}{dt} = kp_5{}^B.[KKP_B**].[KK_B**]$$

$$-(kp_6{}^B + kp_{-5}{}^B).[KKP_B**.KK_B**] \quad (24)$$

$$\frac{d[KK_B**]}{dt} = k_6{}^B.[KKK*.KK_B*] + kp_{-5}{}^B.[KKP_B**.KK_B**] - kp_5{}^B.[KKP_B**].[KK_B**]$$

$$+ (k_{-7}{}^B + k_8{}^B).[KK_B**.K_B] - k_7{}^B.[KK_B**].[K_B]$$

$$+ (k_{-9}{}^B + k_{10}{}^B).[KK_B**.K_B*] - k_9{}^B.[KK_B**].[K_B*] \quad (25)$$

$$\frac{d[K_B]}{dt} = k_{-7}{}^B.[KK_B**.K_B] + kp_8{}^B[KP_B*.K_B*] - k_7{}^B.[KK_B**].[K_B] \quad (26)$$

$$\frac{d[KK_B**.K_B]}{dt} = k_7{}^B.[KK_B**].[K_B] - (k_8{}^B + k_{-7}{}^B).[KK_B**.K_B] \quad (27)$$

$$\frac{d[KP_B*.K_B*]}{dt} = kp_7{}^B.[KP_B*].[K_B*] - (kp_{-7}{}^B + kp_8{}^B).[KP_B*.K_B*] \quad (28)$$

$$\frac{d[K_B*]}{dt} = k_8{}^B.[KK_B*.K_B] + kp_{-7}{}^B.[KP_B*.K_B*] - kp_7{}^B.[KP_B*].[K_B*]$$

$$+ k_{-9}{}^B.[KK_B**.K_B*] - k_9{}^B.[KK_B**].[K_B*] + kp_{10}{}^B[KP_B**.K_B**] \quad (29)$$

$$\frac{d[KK_B**.K_B*]}{dt} = k_9{}^B.[KK_B**].[K_B*] - (k_{-9}{}^B + k_{10}{}^B).[KK_B**.K_B*] \quad (30)$$

$$\frac{d[KP_B**.K_B**]}{dt} = kp_9{}^B.[KP_B**].[K_B**] - (kp_{-9}{}^B + kp_{10}{}^B).[KP_B**.K_B**] \quad (31)$$

$$\frac{d[K_B**]}{dt} = k_{10}{}^B.[KK_B**.K_B*] + kp_{-9}{}^B.[KP_B**.K_B**]$$

$$- kp_9{}^B.[KP_B**].[K_B**] \quad (32)$$
